# APSIC guidelines for disinfection and sterilization of instruments in health care facilities

**DOI:** 10.1186/s13756-018-0308-2

**Published:** 2018-02-20

**Authors:** Moi Lin Ling, Patricia Ching, Ammar Widitaputra, Alison Stewart, Nanthipha Sirijindadirat, Le Thi Anh Thu

**Affiliations:** 1 Infection Prevention & Control, Singapore General Hospital, Singapore, 169608 Singapore; 2 Hong Kong Infection Control Nurses Association (HKICNA), Hong Kong, Hong Kong; 3 Himpunan Sterilasi Sentral Indonesia (HISSI), Jakarta, Indonesia; 4 New Zealand Sterile Services Association (NZSSA), Waikiwi, New Zealand; 5 Central Sterilizing Services Association, Bangkok, Thailand; 6 Ho Chi Minh City Infection Control Society (HICS), Ho Chi Minh City, Vietnam

**Keywords:** Cleaning, Disinfection, Sterilization, Endoscopes, Reprocessing

## Abstract

**Background:**

The Asia Pacific Society of Infection Control launched its revised Guidelines for Disinfection and Sterilization of Instruments in Health Care Facilities in February 2017. This document describes the guidelines and recommendations for the reprocessing of instruments in healthcare setting. It aims to highlight practical recommendations in a concise format designed to assist healthcare facilities at Asia Pacific region in achieving high standards in sterilization and disinfection.

**Method:**

The guidelines were revised by an appointed workgroup comprising experts in the Asia Pacific region, following reviews of previously published guidelines and recommendations relevant to each section.

**Results:**

It recommends the centralization of reprocessing, training of all staff with annual competency assessment, verification of cleaning, continual monitoring of reprocessing procedures to ensure their quality and a corporate strategy for dealing with single-use and single-patient use medical equipment/devices. Detailed recommendations are also given with respect to reprocessing of endoscopes. Close working with the Infection Prevention & Control department is also recommended where decisions related to reprocessing medical equipment/devices are to be made.

**Conclusions:**

Sterilization facilities should aim for excellence in practices as this is part of patient safety. The guidelines that come with a checklist help service providers identify gaps for improvement to reach this goal.

**Electronic supplementary material:**

The online version of this article (10.1186/s13756-018-0308-2) contains supplementary material, which is available to authorized users.

## Background

With increasing use of complex medical equipment in the healthcare setting, the challenge of ensuring adequate cleaning and disinfection of instruments is increasing. Outbreaks or incidents related to inadequate reprocessing of endoscopes have recently highlighted the urgency of ensuring excellence in practices at the sterilization or reprocessing department [1–8]. The intent of this document is to highlight practical recommendations in a concise format designed to assist healthcare facilities at Asia Pacific region in achieving high standards in sterilization and disinfection. This document is a summary of the revised APSIC Guidelines for Disinfection and Sterilization of Instruments in Health Care Facilities developed by the Asia Pacific Society of Infection Control (APSIC) to give the user an overview of its content [http://apsic-apac.org/wp-content/uploads/2017/01/APSIC-Sterilization-guidelines-2017.pdf]. The full APSIC Guidelines for Disinfection and Sterilization of Instruments in Health Care Facilities should be read and used as reference to guide practice.

## Methods

### Review workgroup composition

APSIC convened experts in Infection Prevention and Control and sterilization from Asia Pacific region to revise the APSIC Guidelines for Disinfection and Sterilization of Instruments in Health Care Facilities first published in 2011. The members of this workgroup are the authors of this paper.

### Literature review and analysis

For the APSIC guideline, the workgroup reviewed previously published guidelines and recommendations relevant to each section and performed computerized literature searches using PubMed. Some examples of keywords used in search include sterilization, disinfection, endoscopes, sterilization guidelines, healthcare.

### Process

The workgroup met on 2 occasions as well as discussed via email correspondences to complete the development of the guideline. Criteria for grading the strength of recommendation and quality of evidence are described in Table 1. The draft was then submitted to two external reviewers, APSIC Executive Committee and national Infection Control societies in Asia Pacific. Comments obtained were then reviewed by the workgroup for necessary edits, following which the final copy was circulated for approval and endorsement by the APSIC Executive Committee and national societies from the Asia Pacific region.

**Table 1:** Categories for strength of each recommendation

Categories for strength of each recommendation	
Category	Definition
A.	Good evidence to support a recommendation for use.
B.	Moderate evidence to support a recommendation for use.
C.	Insufficient evidence to support a recommendation for or against use
D.	Moderate evidence to support a recommendation against use.
E	Good evidence to support a recommendation against use.
Categories for quality of evidence on which recommendations are made	
Grade	Definition
I.	Evidence from at least one properly randomized, controlled trial.
II.	Evidence from at least one well-designed clinical trial without randomization, from cohort or case-controlled analytic studies, preferably from more than one centre, from multiple time series, or from dramatic results in uncontrolled experiments.
III.	Evidence from opinions of respected authorities on the basis of clinical experience, descriptive studies, or reports of expert committees.

## Results

### Recommendations for general principles [9–18]

Keeping an instrument safe for patient’s use is the responsibility of all stakeholders. Refer to Table 2 for example of expected responsibility and accountability of each stakeholder.

**Table 2:** Example of stakeholders and their responsibilities

Stakeholders	Responsibility and Accountability
Infection Prevention and Control Department / Unit	• Consultant to CSSD/TSSU on sterilization and disinfection issues • Works closely with CSSD/TSSU on recall events • Conducts regular audits at CSSD/TSSU to ensure compliance with practice standards
Infection Prevention and Control (IPC) Committee	• Reviews and approves the policies and guidelines on sterilization and disinfection of instruments used in the healthcare facility • Receives report on recall events
Central supply sterile department: CSSD,TSSU	• Provide disinfection and sterilization for instruments & medical devices hospitalwide • Ensure healthy workforce in compliance to institution recommendations on staff immunization and checks • Ensures appropriate training for staff to do their work safely and well • Reports recall events to IPC and Quality/Risk Management
Clinical areas e.g. operating room, outpatient clinics, intensive care units, endoscopy centres	• Safe handling of used instruments • Keep used instruments moist with enzymatic cleanser • Safe transportation to CSSD/TSSU for re-processing • Proper storage of sterile items in clinical areas (if any)
Quality / Risk management	• Assists in investigations of recall events

Spaulding Classification System is used in determining appropriate level of reprocessing of patient-care items and equipment. The system classifies a medical device as critical, semi-critical, or non-critical on the basis of risk to patient safety from contamination on a device. The system also established three levels of germicidal activity (sterilization, high-level disinfection, and low-level disinfection) for strategies with the three classes of medical devices (critical e.g. implants, semi-critical e.g. bronchoscopes, and non-critical e.g. stethoscopes) respectively.

Best practices in reprocessing medical equipment/devices must include the following:

- Adequate review by all parties whenever new equipment/devices are being considered for purchase (e.g. Central Sterile Supplies Department [CSSD], Infection Prevention and Control, engineer, etc);
- A centralized area for reprocessing (CSSD) or an area that complies with the requirements for reprocessing;
- Written policies and procedures for reprocessing each type of medical equipment/device including single use items;
- Training of all staff who perform reprocessing at initiation of employment and at least yearly thereafter through yearly competency testing (written and observation);
- Verification of cleanliness, decontamination or sterility and function of the reprocessed equipment/device;
- Continual monitoring of reprocessing procedures to ensure their quality;
- A corporate strategy for dealing with single-use and single-patient use medical equipment/devices;
- Reporting and investigation of medical incidents (e.g. a root cause analysis may be done to identify areas for improvement);
- Management and reporting of safety-related accidents;
- Complete and proper documentation of all reprocessed items for traceability, recall of improperly reprocessed devices and legal purposes.; and
- Procedures to be followed in emergency situations (e.g. utilities shutdowns, compromised packaging, biological indicator (BI) testing failures).

Decisions related to reprocessing medical equipment/devices should be made by a multi-disciplinary Infection Prevention and Control Committee that includes the individuals responsible for purchasing the equipment/device, reprocessing the equipment/device, maintaining the equipment/device, infection prevention and control, occupational health and safety, and the end-user of the equipment/device.

It is strongly recommended that, wherever possible, reprocessing should be performed in a centralized area that complies with the physical and human resource requirements for reprocessing.

1. *Critical medical and surgical devices and instruments that enter normally sterile tissue, body space or vascular system must be sterilized before use. (IA)*
2. *Steam sterilization is the preferred method for sterilizing critical medical and surgical devices and instruments that are not damaged by heat, steam, pressure, or moisture. (IA)*
3. *High-level disinfection is required for semi-critical patient care equipment. (IA)*
4. *Non-critical patient care devices are disinfected when visibly soiled and on regular basis. (IIB)*
5. *Standard sterilization and disinfection procedures are adequate for patient care equipment used on patients with blood-borne pathogens, multidrug resistant organism (MDRO) including multiply resistant Mycobacterium tuberculosis except prions. (IA)*
6. *The following methods are not acceptable for achieving sterilization: (IIIB)*

- *Boiling*
- *Ultraviolet light*
- *Glass bead sterilization*
- *Microwave ovens*
- *Chemiclave sterilization*

7. *Needles must be single-use and must not be reprocessed. (IA)*
8. *The health care setting must have written policies regarding single-use medical equipment/devices. (IIIA)*
9. *Critical and semi-critical medical equipment/devices labelled as single-use must not be reprocessed and re-used unless the reprocessing is done by a licensed reprocessor, which is a facility or unit with legal license to reprocess single use items. (IIA)*
10. *It is strongly recommended that catheters, drains and other medical equipment/devices with small lumens (excluding endoscopy equipment) be designated single-use and not be reprocessed and re-used, even if designated as reusable by the manufacturer. (IIA)*
11. *It is strongly recommended that, wherever possible, reprocessing should be performed in a centralized area that complies with the physical and human resource requirements for reprocessing. (IIIB)*
12. *Persons performing high-level disinfection and/or sterilization should be trained on the science and methods of disinfection/sterilization at initiation of employment and at least yearly. They should undergo competency testing (written and observation at the initiation of employment and at least yearly).*
13. *Persons performing high-level disinfection and/or sterilization should be trained on the proper use of personal protective equipment relevant to the method being used. The health care facility should make available appropriate personal protective equipment.*

### Recommendations for facility design: Environmental requirements for reprocessing areas [19–22]

The CSSD size is appropriately designed with regard to anticipated volume. The central processing area(s) ideally should be divided into at least three areas: cleaning, packaging, and sterilization and storage. Physical barriers should separate the cleaning area from the other sections to contain contamination on used items.

Occupational exposure limits such as ceiling exposure value (CEV) for chemical agents (e.g. glutaraldehyde, ethylene oxide) are to be complied with in accordance to local environmental law.

The health care setting must have air changes; temperature and humidity appropriate to the process/product being used. In health care settings where there are dedicated central reprocessing areas, negative pressure airflow must be maintained in cleaning areas and positive pressure airflow must be maintained in clean areas and be monitored regularly. If monitoring is done centrally externally, the CSSD should be alerted when relative humidity or temperature are out of specified range so that immediate necessary actions can be taken.

1. *The cleaning work area must be physically separated from clean areas by walls or partitions. (IIA)*
2. *Reprocessing performed outside the CSSD must be kept to a minimum and must be approved by the Infection Prevention and Control Committee or those accountable for safe reprocessing practices and must conform to the requirements for reprocessing space. (IIIB)*
3. *Wherever chemical disinfection/sterilisation is performed, air quality must be monitored when using products that produce toxic vapours and mists. (IA)*
4. *There must be a regular schedule for environmental cleaning in the CSSD that includes written procedures and clearly defined responsibilities. (IIB)*

### Recommendations for policies and procedures [23, 24]

Policies and procedures must be established to ensure that the disinfection and sterilization processes follow the principles of infection prevention as set out by CDC, WHO or the country Ministry of Health. Completed policies and procedures should be reviewed and approved by the Infection Prevention and Control Committee. They must be readily accessible to staff doing the reprocessing. Review of reprocessing policies and procedures must take place at least annually**.**

1. *The health care facility will, as a minimum, have policies and procedures for all aspects of reprocessing that are based on current recognized standards/recommendations and that are reviewed at least annually. (IIIA)*
2. *All policies and procedures for reprocessing medical equipment/devices require review and approval by the Infection Prevention and Control Committee. (IIIA)*
3. *Procedures for disinfection and sterilization must include statements and information regarding the type, concentration and testing of chemical products; duration and temperature of exposure; and physical and chemical properties that might have an impact on the efficacy of the process. These procedures must be readily accessible to staff performing the function. (IIA)*
4. *The reprocessing method and products required for medical equipment/devices will depend on the intended use of the equipment/device and the potential risk of infection involved in the use of the equipment/device. (IIIA)*
5. *A procedure should be established for the recall of improperly reprocessed medical equipment/devices. (IIA)*
6. *The recall procedure should include assessment of client/patient/resident risk and a procedure for subsequent notification of physicians, clients/patients/residents, other facilities and/or regulatory bodies if indicated. (IIA) A workgroup may need to be appointed to discuss risk and steps to be taken in the recall process.*
7. *A process for receiving and disseminating medical device alerts and recalls originating from manufacturers or government agencies should be described. (IIIA)*
8. *Products used for any/all stages in reprocessing (*i.e.*, cleaning, disinfection, sterilization) must be approved by the committee responsible for product selection, by an individual with reprocessing expertise and by an individual with infection prevention and control expertise. (IIIA)*
9. *Products used for any/all stages in reprocessing must be appropriate to the level of reprocessing that is required for the use of the medical equipment/device. (IIIA)*
10. *The process and products used for cleaning, disinfection and/or sterilization of medical equipment/devices must be compatible with the equipment/devices. (IIA)*
11. *All medical equipment/devices that will be purchased and will be reprocessed must have written device-specific manufacturer’s cleaning, disinfection and sterilization instruction. If disassembly or reassembly is required, detailed instructions with pictures are highly recommended. Staff training must be provided on these processes before the medical equipment/device is placed into circulation. (IIA)*

### Recommendations for occupational health and safety for reprocessing [25–28]

An Occupational Health and Safety review is recommended for all protocols for reprocessing medical equipment/devices to verify that staff safety measures are followed and are in compliance with the local Occupational Health and Safety Act.

A. *Occupational Health and Safety for the healthcare setting will review all protocols for reprocessing medical equipment/devices to verify that worker safety measures and procedures to eliminate or minimize the risk of exposure are followed and are in compliance with the Occupational Health and Safety Act of the country. (IIA)*
B. *There is a policy that prohibits eating/drinking, storage of food, smoking, application of cosmetics or/and handling contact lenses in the reprocessing area. (IIA)*
C. *Appropriate personal protective equipment (PPE) should be worn for all reprocessing activities. (IA)* See Table 3.
D. *All staff working in reprocessing shall be offered Hepatitis B immunization unless they have documented immunity to Hepatitis B. (IA)*
E. *Measures and procedures shall be written to prevent and manage injuries from sharp objects. (IA)*
F. *Measures and procedures shall be in place for immediate response to worker exposure to blood and body fluids. (IA)*
G. *Health care personnel should also be offered vaccines for vaccine preventable diseases as per institutional policy (*e.g.*, mumps-measles-rubella, varicella, influenza, tetanus-diphtheria or tetanus-diphtheria-acellular pertussis). (IIA)*

**Table 3:** Recommended PPE for CSSD staff working in designated zones for prevention blood and body fluid exposures

CSSD zone	Recommended PPE
Decontamination	Hair covering Water resistant gown Heavy duty disposable gloves Water resistant mask Face shield or goggle
Preparation and packing	Scrubs Hair covering Mask
Sterilization	Scrubs Hair covering Mask
Sterile storage	Scrubs Hair covering Mask
Dispatching	Scrubs Hair covering

Additional PPE should be worn for chemical spills and handling hot objects

### Recommendations for handling and transportation of used medical equipment / devices [9–11, 29–33]

1. *Gross soil should be removed immediately after use by an assigned trained person*. *(IA)*
2. *Disposable components shall be disposed prior to transportation. Disposable sharps shall be disposed of in an appropriate puncture-resistant sharps container at point of use, prior to transportation. (IIA)*
3. *Used items should be kept moist. (IIA)*
4. *Used items must be handled in a manner that reduces the risk of exposure and/or injury to personnel and clients/patients/residents, or contamination of environmental surfaces. (IIA) These may be identified by color code to indicate that these are dirty items.*
5. *Used items should not be transported through high traffic (public) areas, designated areas for storage of clean or sterile supplies, or client/patient/resident care areas. (IIIA)*
6. *Sterile/ clean and used items shall not be transported together. (IIA)*
7. *Transport carts shall be cleaned and dried between uses. There should be a physical barrier between the bottom shelf and the floor. (IIIA)*

### Recommendations for cleaning and verification of reusable medical equipment/devices [32, 34, 35]

Policies and procedures for cleaning medical equipment/devices shall be based on the manufacturer’s instructions and must be developed in consultation with Infection Prevention and Control, Occupational Health and Safety, Biomedical Engineering and Environmental Services. Full PPE shall be worn for handling and cleaning contaminated equipment/devices.

1. *Reusable medical equipment/devices must be thoroughly cleaned prior to before disinfection or sterilization. (IA)*
2. *Factors that affect the ability to effectively clean medical equipment/devices shall be considered prior to cleaning. (IIA)*
3. *Personnel must use appropriate PPE whenever cleaning reusable medical equipment/ devices. (IA)*
4. *The process for cleaning shall include written protocols for disassembly, sorting, soaking, manual or mechanical cleaning, rinsing and drying. (IIIA)*
5. *There shall be a process to ensure that item which have been cleaned can be reliably differentiated from equipment/devices which have not been cleaned (*e.g.*, color coding). (IIIA)*
6. *Products shall be approved by the committee/ team responsible for product selection; by an individual with reprocessing expertise and by infection prevention expertise. (IIIA)*
7. *Products that are used in cleaning process must be compatible with equipment/ device to be reprocessed and used according to manufacturer’s instructions. (IIIA)*
8. *Audits of the cleaning process shall be done on a regular basis. (IIA)*

### Recommendations for instrumentation inspection, Preparation & Packaging [36–38]

After reusable medical equipment/devices have been cleaned they are then inspected, assembled into sets and trays, and packaged for subsequent terminal sterilization. Inspection is required to confirm cleanliness and function. Only packaging materials intended for this use are to be used.

1. *Reusable medical equipment/devices must be thoroughly inspected, prepared before packaging and sterilized ready to use and ensure patient safety. (IIA)*
2. *Effective packaging materials for sterilization should, as a minimum, allow adequate air removal, sterilizing agent penetration, provide an adequate barrier to microorganisms, resist tearing or puncture, provide complete seal and integrity, free of toxic ingredients, non-linting and cost-effective. (IIA)*
3. *Rigid container systems should be cleaned after each use. All components including filters should be disassembled for proper cleaning following manufacturer’s instruction for us [IFU]. (IIA)*

### Recommendations for disinfection of reusable medical equipment / devices [39–42]

When selecting a disinfectant for reprocessing medical equipment/devices in the health care setting, consideration needs to be given to:

- Efficacy for the intended use;
- Compatibility with the equipment/device and surfaces to be disinfected;
- The intended end use of the equipment/devices to be disinfected;
- The method for monitoring the product concentration;
- Recommendations for rinsing following disinfection (e.g., water quality, volume, time);
- Safety for use, with minimal toxic and irritating effects to staff; and
- Environmental safety and biodegradability.

The manufacturer’s recommendations for chemical disinfectants must be followed pertaining to:

- Usage - disinfectant manufacturers must supply recommended usage for the disinfectant to ensure that it is compatible with the medical equipment/devices on which it will be used;
- Contact time (NOTE: where the manufacturer recommends a shorter contact time with a particular product than is required to achieve the desired level of disinfection/sterilization, an infection prevention and control professional must be consulted for advice);
- Use life;
- Proper disposal;
- Storage;
- Appropriate dilution; and
- Required PPE.

1. *Non-critical medical equipment/devices are to be cleaned then disinfected using a low-level disinfectant. (IIA)*
2. *Semi-critical medical equipment/devices require at a minimum, high-level disinfection but sterilization is preferred. (IA)*
3. *The chemical disinfectant used for disinfecting medical equipment/devices must be compatible with both the equipment/device manufacturer’s instructions for disinfection and the cleaning products involved in the reprocessing of the equipment/device. (IA)*
4. *Disinfectant manufacturers must supply recommended usage for the disinfectant to ensure that it is compatible with the medical equipment/devices on which it will be used. (IIIA)*
5. *The process of high-level disinfection requires monitoring and auditing. If a chemical product is used, the concentration of the active ingredient(s) must be verified and a logbook of daily concentration test results is to be maintained. (IA)*
6. *Manufacturer’s instructions for installation, operation and on-going maintenance of hot water pasteurization (often used for respiratory and anesthesia equipment reprocessing) equipment must be followed to ensure that the machine does not become contaminated. (IA)*
7. *A preventive maintenance program for pasteurizing equipment must be implemented and documented. (IIIA)*
8. *Following the pasteurizing cycle, medical equipment/devices shall be thoroughly dried in a drying cabinet that is equipped with a high efficiency particulate air (HEPA) filter and that is used exclusively for the drying of pasteurized equipment/devices. (IIIA)*
9. *A log of contents, temperature and time is to be maintained for each pasteurizer cycle. (IIA)*

### Recommendations for sterilization of reusable medical devices [43, 44]

The sterilization method chosen must be compatible with the item to be sterilized to avoid damage and must be able to achieve 6 log reduction demonstrating sterility assurance. The sterilizer manufacturer’s instructions should be followed for correct loading and operation of individual sterilizers. Chemical and biological methods of monitoring are to be designed for the purpose and stored and used in accordance with the indicator manufacturer’s instructions for use. Sterilization is a process not an event.

Routine monitoring (physical, biological and chemical monitoring) is done to verify the function of sterilizers and the sterilization process. Monitoring is done when a sterilizer is first installed before it is put into general use and to assess routine performance thereafter as recommended in the IFUs. Performance monitoring using all three types of monitors must be completed in all sterilizers to ensure that effective sterilization has been achieved.

Routine monitoring consists of monitoring every package and sterilization load, sterilizer efficacy and periodic product quality assurance testing.

As a minimum the following monitoring should be carried out for loads including medical devices;

- Physical monitoring - includes printouts, digital readings, graphs, gauges to verify the parameters of each cycle have been met. Printouts should be stored safely per institutional policy.
- Chemical monitoring - chemical indicators should be placed on the outside and inside of every package. External indicators (Type 1, Category e) identify processed from non-processed items. Internal indicators (Type 3, 4, 5, 6, Category i) verify the sterilizing agent has reached the contents of the package and critical variables of the process have been met. The variables monitored will depend on the specific type of internal chemical indicator. For loads containing implants, a process challenge device (PCD) containing a biological indicator and Type 5 integrating indicator must be included in each load.
- Biological monitoring – is to be included in the first instrument load of the day and is optional for the remaining loads of the day not containing implants. PCDs containing a BI are required for each load containing implants.

1. *Policies and procedures for sterilizing processes, including loading and unloading the sterilizer, operation of the sterilizer, testing and monitoring, are documented and available. (IIA)*
2. *Sterilizer manufacturer’s written instructions for use are available and loading configurations and cycle parameters are followed. Safety data sheets are available for chemical sterilization. (IIIA)*
3. *Medical device manufacturer’s instructions for use, including sterilizing type and cycle parameters are available, including for loan sets. (IIIB)*
4. *Policies and procedures specific to immediate use steam sterilization (IUSS) are documented and available. Records are maintained, reviewed and demonstrates use of IUSS is restricted and not used for implantable devices. (IIA)*
5. *Procedure for loading shall ensure similar items requiring the same cycle parameters are grouped together. Loading configuration of sterilizer carriages includes;*
6. *Allowing space between packs;*
7. *Carriages are not overloaded;*
8. *Packages do not touch the sterilizer chamber walls;*
9. *Metal items are placed below textiles and pouches;*
10. *Hollow ware,* i.e. *bowls are placed on edge to allow condensate to drain;*
11. *Paper-plastic pouches arranged in a basket on edge or on steriliser carriage with paper side down in a single layer for large items; and*
12. *Rigid containers placed on carriages according to the manufacturer’s recommendations. (IIIB)*
13. *Devices shall be removed from the sterilizer at the completion of the cycle and shall remain on the carriage for at least 30 min or until the outside is cool to the touch. For small sterilizers the load shall be removed from the chamber and placed on a rack to cool. Sterilized devices are cooled in low traffic areas with no air conditioning. (IIIB)*
14. *Sterilisation loads, including IUSS, are documented, results of load indicators recorded and parameters achieved verified and load released for use. Checks made are:*
15. *Parameters verified by reviewing the printout and signing exposure time and cycle completion;*
16. *Bowie Dick test (residual or dynamic air removal test) completed daily;*
17. *Biological monitoring completed at least daily, in every load containing implants and each load for gaseous sterilization methods;*
18. *Internal chemical indicator placed in each package; and*
19. *External indicators achieved correct change (IIA)*
20. *A policy and procedure is in place for the recall of improperly reprocessed medical devices. Records demonstrate adherence to policy and procedure. Policy must include requirement for review of all recalls required. (IIA)*

### Recommendations for release to sterile storage and distribution to point of use [13]

Procedures for the review of records and release of the medical devices from the sterilizing processes are to be specified and documented. The following visual checks need to be completed at a minimum;

- Packaging used is suitable for the sterilizing process and is the correct size for the device sterilized
- The pack is labelled correctly to identify the contents, the seal is intact and the processing chemical indicator has a correct change
- The cycle parameters are achieved and signed as having been checked
- Loads containing biological indicators are quarantined until the results are known and recorded. The load can be released when there is a no growth result on the processed BI PCDs if used, are read and correct
- There is no visible moisture or droplets.

No device should be released if criteria have not been met. The recall policy and procedure must be followed where there are non-conforming sterilized devices.

Information collected as part of the release of sterilized devices should form part of the traceability records so that the patient can be tracked back to the process. IUSS devices should be released in the same way as devices going through the usual processes.

1. *Written policies and procedures are available for storage, handling, rotation and labelling of sterile packs. (IIA)*
2. *Reprocessed medical devices shall be stored in a clean, dry location in a manner that minimizes contamination or damage. Traffic in the sterile storage area is controlled to limit access; no external shipping cartons are present. Shelving is at least 20 – 25 cm above the floor, at least 45 cm from the ceiling or sprinkler heads, and at least 5 cm from outside walls. Supplies are only stored on designated shelving, counter and carts (not on windowsills, floors* etc.*). (IIA)*
3. *Sterile storage area is generally ≤ 24 °C, relative humidity does not exceed 70%, minimum air changes per hour of 4 downward-draft type. (IIB)*
4. *Rotation of stock is maintained on a first in first out (FIFO) basis. (IIA)*
5. *At point of use, upon opening the reprocessed medical device, check for integrity of the packaging and the device; validate results of chemical monitors if present; and reassemble device if required. (IIA)*

### Recommendations for calibration and maintenance of reprocessing equipment [13]

Instrumentation used to control or monitor reprocessing equipment, e.g. timers, gauges and temperature monitoring devices, shall be recalibrated regularly to prove their accuracy, at least annually and immediately prior to requalification.

Preventative maintenance should be carried out in accordance with the equipment manufacturer’s instructions for use. To achieve this, a qualified individual should carry out maintenance of the equipment. Particular attention should be given to inspection, maintenance and replacement of components subject to normal wear and tear such as recording devices, filters, steam traps, drain pipes, valves and door gaskets. A schedule for maintenance and the work carried out shall be maintained for each piece of reprocessing equipment.

1. *A schedule and maintenance record is kept and available for each piece of reprocessing equipment. These demonstrate planned preventive maintenance is being undertaken according to the equipment manufacturer’s instructions for use. (IIIA)*
2. *Calibration of instruments used to control and monitor the equipment is carried out periodically according to the equipment manufacturer’s instructions for use and at other times where a replaced component requires it. (IIIA)*
3. *A qualification test is to be done after new installation, relocation, major repairs and any other environmental changes. The process must be fully documented, all test results documented and the documentation reviewed. (IIA)*

### Recommendations for reprocessing endoscopy equipment/devices [45–55]

Due to the complexity of their design, flexible fibreoptic and video endoscopes (‘semi-critical endoscopes’) require special cleaning and handling. Since flexible bronchoscopes and cystoscopes are entering a sterile cavity, it is highly recommended that these be sterilized; however, if they are not compatible with sterilization, high-level disinfection is the minimum requirement.

Individuals responsible for reprocessing endoscopes require training and must meet the health care facilities written endoscope processing competency requirements, which include on-going education and training.

To minimize the immediate risk, it is recommended to adhere to current endoscope reprocessing guidelines where pre-cleaning is done with aim to decrease organic load especially at the elevator with any one of the following methods for reprocessing duodenoscopes (priority ranked):

- Ethylene oxide sterilization after HLD with periodic microbiologic surveillance
- HLD done twice with periodic microbiologic surveillance
- HLD with scope quarantine until negative culture
- Liquid chemical sterilant processing system using peracetic acid (rinsed with extensively treated potable water) with periodic microbiologic surveillance
- Other FDA-cleared low-temperature sterilization technology (provided material compatibility and sterilization validation testing performed using the sterilizer and endoscope) after HLD, with periodic microbiologic surveillance
- HLD with periodic microbiologic surveillance

1. *Individuals responsible for reprocessing endoscopes shall be specially trained and shall meet the facility’s written endoscope processing competency requirements, including ongoing education and training and annual competency testing (IA)*
2. *Each health care setting in which endoscopic procedures are performed shall have written, detailed procedures for the cleaning and handling of endoscopes. (IIA)*
3. *Critical endoscopes shall be sterilized prior to use. (IA)*
4. *Semi-critical endoscopes require a minimum of high-level disinfection prior to use. (IA)*
5. *Adequate ventilation is required to remove toxic vapours generated by, or emitted from, cleaning or disinfecting agents. (IA)*
6. *Endoscope cleaning shall commence immediately following completion of the clinical procedure. (IA)*
7. *Patency and integrity of the endoscope sheath shall be verified through leak testing, performed after each use. (IA)*
8. *Endoscopic equipment/devices shall be rinsed and excess water removed prior to disinfection or sterilization. (IIA)*
9. *Endoscopic accessories (*e.g.*, biopsy forceps and brushes) that enter sterile tissue or the vascular system shall be disposable or sterilized after each use. (IA)*
10. *Final drying of semi critical endoscopes shall be facilitated by flushing all channels with filtered air, followed by 70% isopropyl alcohol, followed by forced air purging of the channels. (IA)*
11. *Semi critical endoscopes shall be stored in a dedicated, closed, ventilated cabinet outside of the reprocessing area and procedure room. (IIA)*
12. *The water bottle and its connecting tube, used for cleaning the endoscope lens and irrigation during ERCP (endoscopic retrograde cholangiopancreatography) procedures, shall be cleaned and sterilized following manufacturer’s instructions. (IIIA)*
13. *A preventive maintenance program for automated endoscope reprocessor (AER) shall be implemented and documented. (IIIA)*
14. *Healthcare settings shall have policies in place providing a permanent record of endoscope use and reprocessing, as well as a system to track endoscopes and clients/patients/residents that includes recording the endoscope number in the client/patient/resident record. (IIIA)*
15. *Enhancement in methods for reprocessing duodenoscopes should be followed and documented. (IIA)*
16. *Regular surveillance for bacterial contamination of duodenoscopes after reprocessing using a special culture method and test is recommended. (IIA)*

### Recommendations for education and training [19]

The manager and all supervisors involved in reprocessing must, as a minimum, have completed a recognized qualification/certification course in reprocessing practices. A plan must be in place for each person involved in reprocessing to obtain this qualification. It is strongly recommended that continuing education and or re-certification be obtained at a regular interval. All staff involved in reprocessing of medical equipment/devices must be supervised and shall be qualified through education in a formally recognized course for sterilization technology, training and experience in the functions they perform shall be provided at regular intervals and periodic competency assessment all orientation, training and continuing education is documented.

1. *The policies of the healthcare setting shall specify the requirements for, and frequency of, education and training as well as competency assessment for all personnel involved in the reprocessing of medical equipment/devices. (IIA)*
2. *All aspects of reprocessing shall be supervised and shall be performed by knowledgeable, trained personnel. (IIA)*
3. *Managers, supervisors and staff involved in reprocessing have completed a recognized qualification/certification course in reprocessing practices. (IIA)*
4. *A plan must be in place for each person involved in reprocessing to obtain certification qualification. (IIIA)*

## Conclusion

We recommend sterilization facilities to aim for excellence in practices in following aspects:

1. Handling, collection and transport of contaminated instruments
2. Cleaning and decontamination processes
3. Instrumentation inspection, preparation & packaging
4. Sterilisation and monitoring
5. Sterile storage and distribution
6. Documentation
7. Facility Design

A checklist for self-assessment is included in the guidelines to help service providers identify gaps for improvement in the above aspects. The APSIC CSSD Centre for Excellence Award is run by APSIC where the aim is to promote excellence in compliance to standards for sterilization practices at the CSSD in hospitals at the Asia Pacific region. A CSSD is deemed as a Center of Excellence when it fulfills the criteria listed in the APSIC Self-Assessment Framework (see Additional file 1: Appendix). The center not only delivers quality disinfection and sterilization services but is also committed to education and research and takes a leadership role to help and support other CSSD institutions in their implementation of CSSD education programs.

Endorsed by:

1. Borneo Infection Control Society
2. New Zealand Sterile Sciences Association (NSSA)
3. Infection Control Association of Singapore (ICAS)
4. Ho Chi Minh City Infection Control Society, Vietnam
5. The Central Sterilization Services Association of Thailand (CSSA)
6. The Thai Perioperative Nurses Association (TPNA)
7. The Nursing Association for Prevention and Control of Infections (NAPCI)
8. Central Services and Sterilization Association of the Philippines (PACSSM)
9. Korea Association of Central Supply Department Nurses (KACSDN)
10. Hong Kong Infection Control Nurses Association (HKICNA)

## Additional file

Additional file 1: Appendix. (DOCX 54 kb)
