# Supplementary material for: APSIC guidelines for disinfection and sterilization of instruments in health care facilities
Source: Antimicrob Resist Infect Control. 2018 Feb 20;7:25. doi: 10.1186/s13756-018-0308-2 (PMC5819152; doi:10.1186/s13756-018-0308-2)
Supplement: Supplementary file 1 — Appendix. (DOCX 54 kb) [file 13756_2018_308_MOESM1_ESM.docx]

**Appendix**

**APSIC Checklist**

Note: Yellow highlighted boxes refer to mandatory items

**(A) Handling, Collection and Transport of Contaminated Instruments**

|  | **Item reviewed** | **Yes/No** | **Action Plan** |
| --- | --- | --- | --- |
| 1 | Reusable items separated from waste at point of use |  |  |
| 2 | Contaminated disposable items are discarded appropriately (including sharps.) |  |  |
| 3 | Gross soil is removed from instruments at point of use if immediate transportation not possible |  |  |
| 4 | Soiled items should be kept moist (moist towel, enzyme foam or spray product) |  |  |
| 5 | Secured, dedicated containers are provided for soiled instruments |  |  |
| 6 | Use of puncture resistant, leak-proof containers for soiled items |  |  |
| 7 | Soiled items must be contained during transportation |  |  |
| 8 | Transportation of soiled instruments avoids high (public) traffic areas |  |  |
| 9 | Transportation carts should be covered and should prevent items from falling over or off |  |  |
| 10 | Dedicated elevators (or lifts) with direct access to decontamination area. |  |  |
| 11 | Policy and procedure in place for transportation of contaminated items between buildings, if applicable |  |  |

**(B) Cleaning and Decontamination Processes**

|  | **Item reviewed** | **Yes/No** | **Action Plan** |
| --- | --- | --- | --- |
| 1 | Written policies and procedures in place for all cleaning and decontamination processes. |  |  |
| 2 | Instrumentation is disassembled (according to manufacturer’s instructions) to expose all surfaces for cleaning |  |  |
| 3 | Rigid container systems disassembled according to manufacturer instructions (filters, valves and interior baskets.) |  |  |
| 4 | Cleaning agents are used according to manufacturer’s instructions (dilution and temperature, etc.) |  |  |
| 5 | Appropriate manual and mechanical cleaning methods are used according to manufacturer’s instructions and IFU’s are accessible to decontamination staff |  |  |
| 6 | Appropriate personal protective equipment (PPE) are used |  |  |
| 7 | Appropriate brushes/cleaning implements designed for use on medical devices are used. |  |  |
| 8 | Brushes/cleaning implements are either disposable or if reusable, are decontaminated at least daily. |  |  |
| 9 | Monitoring of mechanical cleaning equipment should be done upon installation and then weekly (preferably daily) and recorded |  |  |
| 10 | Appropriate manual and mechanical rinsing methods are understood and are done according to manufacturer’s instructions. |  |  |
| 11 | Cleaning Agent (Enzymatic cleaner) should be compatible with the medical device to be cleaned. |  |  |
| 12 | Chemical for disinfectants and terminal sterilisation are used according to manufacturer’s instructions |  |  |
| 13 | Ultrasonic cleaner solution is changed at specified frequency or sooner if needed |  |  |
| 14 | Final rinse in washer disinfector is done with treated water (deionized, distilled, or RO water) |  |  |

**(C) Instrumentation Inspection, Preparation & Packaging**

|  | **Item reviewed** | **Yes/No** | **Action Plan** |
| --- | --- | --- | --- |
| **Instrument inspection** | | | |
| 1 | Ensure instruments are cleaned and dried before packaging. |  |  |
| 2 | Inspect instruments for flaws or damage. Check for rust, pitting, corrosion, burrs, nicks, cracks, chipping of plated surfaces. Lighted magnifying glass available for instrument inspection. |  |  |
| 3 | Cleaning verification by users should include visual inspection combined with other verification methods (ATP) that allow assessment of instrument surfaces and channel |  |  |
| 4 | Instruments: Ensure that   - Cutting edges are sharp; - Moving parts move freely, without sticking. - Instruments needing repair are taken out of service for repair or replacement. |  |  |
| 5 | Follow MDMs instructions for instruments requiring lubrication after cleaning or prior to sterilisation. |  |  |
| **Preparation and Assembly:** | | | |
| 6 | Delicate/sharp instruments are protected while being handled/assembled for sterilisation. (*May use special holders, tip guards, or foam sleeves*).   - Tip protectors should be sterilant-permeable. |  |  |
| 7 | Instruments that open (e.g. scissors, haemostats) are held in unlocked, open positions. |  |  |
| 8 | Multi-part instruments are disassembled prior to sterilisation, ensuring all parts are easily accessed for aseptic assembly. |  |  |
| 9 | Lumened devices:   - Remove stylets/plugs, such as catheters, needles, tubings. - Moistening of the lumen may be recommended; consult device manufacturer. |  |  |
| 10 | Complex instruments (air-powered, endoscopes, having lumens or channels) are prepared according to written instructions from device manufacturer. |  |  |
| 11 | Non-linting absorbent material may be placed in trays to help facilitate drying. Tray liners or other absorbent materials may be used to alleviate drying problems. |  |  |
| 12 | Basins:   - Graduated basins should differ in diameter by one inch. - Use non-linting absorbent material between nested basins. - Wrapped basin sets should not exceed 3 Kg (7 lbs.). |  |  |
| 13 | Containerized instrument sets do not exceed 11kg (25 lbs.). |  |  |
| **Packaging:** | | | |
| 14 | Packaging materials are held for a min. of 2 hrs. prior to use at room temp (21°F-24°F) and at a relative humidity ranging from 30-60%. [*This is needed to permit steam sterilisation and prevent superheating*.] |  |  |
| 15 | Packaging materials are examined regularly for defects (i.e. holes, warn spots, stains). |  |  |
| 16 | Wrappers should be kept snug, but not wrapped too tightly or strike-through could occur. |  |  |
| 17 | Paper/Plastic Pouches:   - Labelling is done on plastic side only. - Double peel pouch only if pouch is validated for this use. |  |  |
| 18 | Wrapped packs: write only on indicator tape or affixed labels. |  |  |
| 19 | Perforated, wire-mesh-bottom trays, and rigid organizing trays are inspected prior to each use to ensure there are no sharp edges, nicks, or loose wire-mesh. |  |  |
| 20 | Tape (other than sterilisation indicator tape) should not be used to secure packages, nor should safety pins, ropes, paper clips, staples, or other sharp objects |  |  |
| 21 | Validation test to be done for heat sealer at set frequency. |  |  |

**(D) Sterilisation and Monitoring**

|  | **Item reviewed** | **Yes/No** | **Action Plan** |
| --- | --- | --- | --- |
| **Follow Manufacturers’ Guidelines:** | | | |
| 1 | Steriliser Manufacturers’ written instructions for cycle parameters are available. |  |  |
| 2 | Rigid Container Manufacturers’ instructions for cycle parameters are followed. |  |  |
| 3 | Medical Device Manufacturers’ written instructions for sterilisation cycle parameters are available/accessible for items to be sterilised, including Loaner sets |  |  |
| **Loading the Steriliser:** (Follow steriliser Mfrs. Written instructions) | | | |
| 4 | Group together similar items requiring same cycle parameters. |  |  |
| 5 | Steriliser Cart:   - Allow space between packs - Do not overload - Packages should not touch chamber walls |  |  |
| 6 | Mixed Loads - Place metal items on the loading cart below textiles and paper-plastic pouches (to prevent condensate from dripping onto lower packs). |  |  |
| 7 | Solid-bottom pans, bowls, and trays are tilted on edge and oriented in the same direction. |  |  |
| 8 | Paper-plastic pouches – Use baskets to facilitate placing pouches on edge. |  |  |
| 9 | Rigid Containers: Stacking could interfere with air evacuation; follow container Manufacturer’s Instructions. |  |  |
| **Unloading the Steriliser:** | | | |
| 10 | Open steriliser door properly.   - Door may be opened slightly at the end of the cycle (for some time) prior to removing the load. |  |  |
| 11 | Load contents: There should be no visible signs of liquid, or water droplets. (Wet items are considered contaminated even if not touched.) |  |  |
| 12 | Sterilised items remain on the cart to cool for a minimum of 30 minutes, and are not touched during the cooling process. |  |  |
| 13 | Place cart in a low traffic area without proximity to air-conditioning or cold-air vents. |  |  |
| 14 | Immediate Use “Flash” Items: Are used immediately and not stored for later use. (Assume condensate will be present.) |  |  |
| **Physical monitors, Chemical indicators, Biological indicators:** | | | |
| 15 | Verify parameters of the cycle have been met by reviewing cycle printout tapes. Circle minimum temperature and exposure time, initial/sign, and date. |  |  |
| 16 | Bowie-Dick Testing is done daily in pre-vacuum sterilisers before first processed load.  Process Bowie-Dick at 132°-134ºC for 3.5 to 4 minutes. One pack per load in an empty chamber. Record results. |  |  |
| 17 | External process indicators (indicator tape, labels) are affixed to hospital-sterilised packages and containers. |  |  |
| 18 | Internal chemical indicator(s) (Type 4, 5, 6) are placed inside every package in the most challenging location for sterilant to reach. (Refer to Rigid Container Manufacturers’ Instructions for CI placement). |  |  |
| 19 | ***Implant Loads:*** Monitor with a BI PCD containing a Type 5 Integrating Indicator.  Implants should be quarantined until BI results are known, except in emergency situations |  |  |
| 20 | ***Non-Implant Loads:*** *Optional* monitoring with a PCD containing either: a BI, a BI and Type 5, a Type 5 integrating indicator, or a Type 6 emulating indicator. |  |  |
| 21 | Routine steriliser efficacy testing with a BI PCD is done daily (if steriliser run daily):  Sterilisers larger than 60 L –Place BI PCD in first load of items to be sterilised, on bottom shelf of steriliser cart over drain.  Table Top sterilisers: BI PCD is run with first load of the day and generally placed in centre of load. |  |  |
| **Use appropriate BI PCD depending on type of steriliser:** | | | |
| 22 | Steam: daily (each day the sterilizer is used) |  |  |
| 23 | Gaseous sterilization (e.g. EO, H_2_O_2_): BI should be used every load |  |  |
| 24 | Sterilisers larger than 60 Litter. Use commercially available FDA-cleared BI PCD or AAMI 16-towel pack. (M) |  |  |
| 25 | Table Top sterilisers. BI PCD is a user assembled challenge test pack, which creates the greatest challenge (e.g., BI in peel pouch, BI in wrapped set) and contains items normally processed. |  |  |
| **BI Test/Control, and Results:** | | | |
| 26 | Control BI: Incubate a positive BI control each day a test vial is incubated and in each Auto-reader or incubator. The Control BI needs to be from the same lot number as the Test BI. Record results. |  |  |
| 27 | Test BI: Incubate Test BI according to BI Manufacturers’ Instructions. Record results. |  |  |
| **Qualification Testing:** | | | |
| 28 | For sterilisation process failures where the cause is not immediately identifiable, and after major steam or steriliser repairs, run 3 empty cycles with a BI PCD followed by 3 empty cycles with a Bowie-Dick test if prevacuum steriliser. |  |  |
| **Steriliser Maintenance:** | | | |
| 29 | Steriliser “drain strainers” are inspected daily for debris. |  |  |
| 30 | Steriliser external and internal surfaces are routinely cleaned. |  |  |

**(E) Sterile Storage and Distribution**

|  | **Item reviewed** | | **Yes/No** | | **Action Plan** |
| --- | --- | --- | --- | --- | --- |
| **STERILE STORAGE:** | | | | | |
| 1 | Written Policies and Procedures are available for storage, handling, rotation, and labelling of sterile packs. |  | |  | |
| 2 | Traffic in the sterile storage area is controlled to limit access to sterile items. |  | |  | |
| 3 | Outside shipping containers and corrugated cartons are not used as containers in sterile storage areas. |  | |  | |
| 4 | Storage area temperature is generally less than 24°C and Relative Humidity should not exceed 70%. |  | |  | |
| 5 | Sterile items are stored at least 20-25cm (8-10”) above the floor, at least 45cm (18”) below the ceiling or sprinkler heads, and at least 5cm (2”) from outside walls. |  | |  | |
| 6 | Shelving and storage carts have a physical barrier between the bottom shelf and the floor. |  | |  | |
| 7 | Medical/Surgical items, including rigid containers, are not stored next to or under sinks, under exposed water/sewer pipes, or in any location where they may become wet. |  | |  | |
| 8 | Supplies are stored only on designated shelving, counters, and carts (not on windowsills, floors, etc.) |  | |  | |
| 9 | When stacking container systems, ensure they are firmly seated on one another. |  | |  | |
| **DISTRIBUTION:** | | | | | |
| 10 | Supplies are distributed on a First In First Out (FIFO) basis |  | |  | |
| 11 | Packaging is inspected visually for integrity, and labelling, prior to using items. |  | |  | |
| 12 | Transport carts should have a physical barrier between the bottom shelf and the floor.   - Reusable covers should be cleaned after each use. |  | |  | |
| 13 | Carts are decontaminated/ dried before reused for transporting sterile supplies. |  | |  | |

**(F) Documentation**

|  | **Item reviewed** | | **Yes/No** | | **Action Plan** |
| --- | --- | --- | --- | --- | --- |
| **EQUIPMENT & CYCLE DOCUMENTATION:** | | | | | |
| 1 | Documentation for each mechanical washer is maintained: Monitor and verify cleaning processes (e.g. digital readouts, and cycle printouts) |  | |  | |
| 2 | Documentation for each steriliser is maintained, and includes results from each load. (e.g. monitoring results; steriliser repair records) |  | |  | |
| 3 | For each cycle printout tape:   - Verify cycle start was initiated - Ensure cycle selected was appropriate for load contents - Verify correct Time & Temp. was met - Ensure there were no cycle aborts or warnings |  | |  | |
| 4 | Record for each cycle:   - Lot number; - Load contents; - Exposure time/temp; *Name/initials of steriliser operator; - Results of BI testing, if applicable; - Results of Bowie-Dick testing, if applicable; - Results of CIs in test packs; reports of non-conclusive or non-responsive CIs found in the load |  | |  | |
| 5 | An instrument tracking system or other type of computer system is used. |  | |  | |
|  | **PRODUCT RECALLS:** |  | |  | |
| 6 | - Policies & Procedures are clear and concise - Records are maintained - Lot control labels are used, to include: Steriliser ID, lot number, sterilisation date, expiration date, name of pack and initials. |  | |  | |
| 7 | Sterilisation Process Failure: – When cannot immediately identify cause of failure (e.g. selected incorrect cycle setting), reprocess the load and recall/reprocess all items dating back to last load in steriliser with negative BI results. |  | |  | |

**(G) Facility Design**

|  | **Item reviewed** | **Yes/No** | **Action Plan** |
| --- | --- | --- | --- |
| 1 | All instrumentation reprocessing is centralized |  |  |
| 2 | If centralized reprocessing is not possible, consistent policies and procedures between locations are in place. |  |  |
| 3 | CSSD department size is appropriately designed with regard to anticipated volume |  |  |
| 4 | Decontamination area facilitates proper workflow and provides adequate space for necessary equipment |  |  |
| 5 | Decontamination area has space dedicated to donning and removal of PPE. |  |  |
| 6 | Decontamination sink is of adequate size and has three compartments (for soaking, cleaning and rinsing) |  |  |
| 7 | Handwashing sinks/hand hygiene facilities are appropriately located in department |  |  |
| 8 | Emergency eyewash stations (required by OSHA) located within 10 seconds travel time of all chemical usage locations, with a continuous flush for at least 15 minutes. E.g., Decontamination area. |  |  |
| 9 | Functional workflow pattern: clear distinction (i.e. physical wall) between dirty and clean |  |  |
| 10 | Functional workflow pattern: pass–through window available to avoid hallways, and is not propped open |  |  |
| 11 | Temperature and humidity monitoring controls in decontamination and clean areas |  |  |
| 12 | Temperature and humidity monitoring is recorded daily |  |  |
| 13 | Appropriate traffic control. Written policy and procedure in place for authorized entry and movement and attire. |  |  |
| 14 | Floors and walls are constructed from materials that can withstand frequent cleaning |  |  |
| 15 | Ceilings are flush surfaces and not of materials that are of a particulate or fibre-shedding composition. |  |  |
| 16 | Doors close freely and do not have thresholds. |  |  |
| 17 | Appropriate positive (clean areas) and negative (soiled areas) pressure ventilation systems in place |  |  |
| 18 | Appropriate air-change in decontamination and storage area |  |  |
| 19 | Lighting adequate for all work areas |  |  |

**(H) Considerations**

|  | **Item reviewed** | **Yes/No** | **Action Plan** |
| --- | --- | --- | --- |
| 1 | CS supervisory personnel meet minimum recommended qualifications |  |  |
| 2 | CS supervisory personnel maintain competency and participate in departmental continuing education |  |  |
| 3 | CS technicians meet minimum recommended qualifications |  |  |
| 4 | All new CS personnel receive initial and comprehensive facility and department orientation. |  |  |
| 5 | All CS personnel receive a minimum annual training on department policies and procedures All CS personnel demonstrate competency annually. |  |  |
| 6 | Written policy on personal hygiene. |  |  |
| 7 | Written policy and adherence to appropriate CS personnel attire. |  |  |
| 8 | Written policy and adherence to appropriate PPE in decontamination area. |  |  |
| 9 | Written policy and schedule for housekeeping |  |  |
| 10 | Written policy and schedule for instrument and sterilizer machine maintenance |  |  |
| 11 | Products used for any/all stages in reprocessing (cleaning, disinfection, sterilization) must be approved by the committee responsible for product selection, by an individual with reprocessing expertise and by an individual with infection prevention and control expertise |  |  |
